# Supplementary material for: 3-Vinylazetidin-2-Ones: Synthesis, Antiproliferative and Tubulin Destabilizing Activity in MCF-7 and MDA-MB-231 Breast Cancer Cells
Source: Pharmaceuticals (Basel). 2019 Apr 11;12(2):56. doi: 10.3390/ph12020056 (PMC6630832; doi:10.3390/ph12020056)
Supplement: Supplementary file 1 [file pharmaceuticals-12-00056-s001.pdf]

Supplementary Information

3-Vinylazetidin-2-ones: Synthesis, antiproliferative and tubulin destabilizing activity in MCF-7 and MDA-MB-231 breast cancer cells

Shu Wang[a], Azizah M. Malebari[a,b*], Thomas F. Greene[a*], Niamh M. O'Boyle[a], Darren Fayne[c], Seema M. Nathwani[c], Brendan Twamley[d], Thomas McCabe[d], Niall O. Keely[a], Daniela M. Zisterer[c], Mary J. Meegan[a**]

[a] School of Pharmacy and Pharmaceutical Sciences, Trinity College Dublin, Trinity Biomedical Sciences Institute, 152-160 Pearse Street, Dublin 2, Ireland

[b] Department of Pharmaceutical Chemistry, College of Pharmacy, King Abdulaziz University, Jeddah, Saudi Arabia

[c] School of Biochemistry and Immunology, Trinity College Dublin, Trinity Biomedical Sciences Institute, 152-160 Pearse Street, Dublin 2, Ireland

[d] School of Chemistry, Trinity College Dublin, Dublin 2, Ireland

Tel: +353-1-8962798; Fax: +353-1-8962793

*Authors contributed equally to the work

**Corresponding author:
Mary J. Meegan
E-mail: mmeegan@tcd.ie

Supplementary Information

Experimental section: Experimental details for preparation and characterisation of intermediate imine compounds **5a-i, 5m-s, 6a-c, 6f-k** …………………………….. 3
Table S1: Tier-1 Profiling Screen of Selected β-Lactams...11
Table S2: ADMET and Lipinski Properties for Selected β-Lactams………………..13
Table S3: Comparative Antitumour Evaluations of compounds **7h, 7s, 7t, 17b, 17c** in the NCI60 *Leukemia, Non-Small Cell Lung Cancer*, *Colon Cancer* and *CNS Cancer* cell lines *in vitro* primary screen…………………………………………….....15
Table S4: Comparative Antitumour Evaluations of compounds **7h, 7s, 7t, 17b, 17c** in the NCI60 *Melanoma*, *Ovarian cancer*, *Renal cancer* and *Breast cancer* cell line *in vitro* primary screen……………………………………………………………… 16
Table S5: NCI 60 cell line mean screening results for selected compounds………..17
Table S6: Standard COMPARE analysis of β-lactam **7h**………………………..….18
Table S7: Standard COMPARE analysis of β-lactam **7s**………………………….. 19
Table S8: Toxicity at 10 μM against two concentrations of murine epithelial cells. 20
Table S9. Toxicity at IC_{50} against two concentrations of murine epithelial cells.......20
References ………………………………………………………………… 21

Experimental section: Experimental details for preparation and characterisation of intermediate imine compounds 5a-i, 5m-s, 6a-c, 6f-k

Materials and methods: Chemistry

All reagents were commercially available and were used without further purification unless otherwise indicated. Tetrahydrofuran (THF) was distilled immediately prior to use from Na/Benzophenone under a slight positive pressure of nitrogen, toluene was dried by distillation from sodium and stored on activated molecular sieves (4Å) and dichloromethane was dried by distillation from calcium hydride prior to use. Uncorrected melting points were measured on a Gallenkamp SMP 11 melting point apparatus. Infra-red (IR) spectra were recorded as thin film on NaCl plates, or as potassium bromide discs on a Perkin Elmer FT-IR Spectum 100 spectrometer. ^{1}H and ^{13}C nuclear magnetic resonance (NMR) spectra were recorded at 27°C on a Brucker Avance DPX 400 spectrometer (400.13 MHz, ^{1}H; 100.61 MHz, ^{13}C) at 20 °C in either $CDCl_3$ (internal standard tetramethylsilane TMS) or CD_3OD by Dr. John O'Brien and Dr. Manuel Ruether in the School of Chemistry, Trinity College Dublin. For $CDCl_3$, ^{1}H-NMR spectra were assigned relative to the TMS peak at 0.00 δ and ^{13}C-NMR spectra were assigned relative to the middle $CDCl_3$ triplet at 77.00 ppm. For CD_3OD, ^{1}H and ^{13}C-NMR spectra were assigned relative to the centre peaks of the CD_3OD multiplets at 3.30 δ and 49.00 ppm respectively. Electrospray ionisation mass spectrometry (ESI-MS) was performed in the positive ion mode on a liquid chromatography time-of-flight (TOF) mass spectrometer (Micromass LCT, Waters Ltd., Manchester, UK) equipped with electrospray ionization (ES) interface operated in the positive ion mode at the High Resolution Mass Spectrometry Laboratory by Mr. Brian Talbot in the School of Pharmacy and Pharmaceutical Sciences, Trinity College Dublin and Dr. Martin Feeney in the School of Chemistry, Trinity College Dublin. Mass measurement accuracies of < ±5 ppm were obtained. Low resolution mass spectra (LRMS) were acquired on a Hewlett-Packard 5973 MSD GC-MS system in electron impact (EI) mode. R_f values are quoted for thin layer chromatography on silica gel Merck F-254 plates, unless otherwise stated. Flash column chromatography was carried out on Merck Kieselgel 60 (particle size 0.040-0.063 mm). Chromatographic separations were also carried out on Biotage SP4 instrument. All products isolated were homogenous on TLC. Analytical high-performance liquid chromatography (HPLC) to determine the purity of the final

compounds was performed using a Waters 2487 Dual Wavelength Absorbance detector, a Waters 1525 binary HPLC pump, a Waters In-Line Degasser AF and a Waters 717plus Autosampler. The column used was a Varian Pursuit XRs C18 reverse phase 150 x 4.6 mm chromatography column. Samples were detected using a wavelength of 254 nm.

Experimental section

General method I: Preparation of imines 5a-I, 5m-s, 6a-c, 6f-k

The appropriately substituted benzaldehyde (10 mmol) and corresponding substituted aniline (10 mmol) were heated reflux in ethanol (40 mL) for 4 h with a catalytic amount of concentrated sulphuric acid. The volume of reaction was then reduced to approximately 10 mL in *vacuo*. The Schiff base precipitated from solution upon standing at room temperature overnight. The solid product obtained was filtered and purified by recrystallisation from ethanol.

(*E*)-1-(4-Fluorophenyl)-*N*-(3,4,5-trimethoxyphenyl)methanimine (5a). Preparation as described above from 4-fluorobenzaldehyde and 3,4,5-trimethoxyaniline. The product was obtained as a colorless solid, yield 81%, Mp: 100-102 ˚C.[1] IR (KBr) ν_{max}: 1638 (C=N) cm^{-1}. ^1H NMR (400 MHz, $CDCl_3$): δ 3.84 (s, 3H), 3.93 (s, 6H), 6.51 (s, 2H), 7.17-7.22 (m, 2H), 7.91-7.94 (m, 2H), 8.47 (s, 1H). ^{13}C NMR (100 MHz, $CDCl_3$): δ 55.93, 61.05, 98.13, 115.92, 130.73, 130.82, 132.23, 132.32, 147.69, 153.61, 158.22, 163.49. HRMS: found 290.1192 (M^++H); $C_{16}H_{17}FNO_3$ requires 290.1192.

(*E*)-1-(4-Chlorophenyl)-*N*-(3,4,5-trimethoxyphenyl)methanimine (5b). Preparation as described above from 4-chlorobenzaldehyde and 3,4,5-trimethoxyaniline. The product was obtained as pale yellow solid, yield 75%, Mp 110-112 ˚C. [2] IR (KBr) ν_{max}: 1627 (C=N) cm^{-1}. ^1H NMR (400 MHz, $CDCl_3$): δ 3.89 (s, 3H), 3.93 (s, 6H), 6.53 (s, 2H), 7.48 (d, J = 8.52 Hz), 7.88 (d, J = 8.52 Hz), 8.46 (s, 1H). ^{13}C NMR (100 MHz, $CDCl_3$): δ 55.69, 60.58, 97.71, 128.67, 129.45, 130.47, 134.06, 136.96, 147.05, 153.15, 157.71. HRMS: found 306.0903 (M^++H); $C_{16}H_{17}{}^{35}ClNO_3$ requires 306.0897.

(*E*)-1-(4-Bromophenyl)-*N*-(3,4,5-trimethoxyphenyl)methanimine (5c). Preparation as described above from 4-bromobenzaldehyde and 3,4,5-trimethoxyaniline. The product was obtained as pale yellow solid, yield 70%, Mp 110-112 ˚C [3] IR (KBr) ν_{max}: 1624 (C=N) cm^{-1}. ^1H NMR (400 MHz, $CDCl_3$): δ 3.89 (s, 3H), 3.92 (s, 6H), 6.51 (s, 2H), 7.63 (d, J = 8.52 Hz, 2H), 7.79 (d, J = 8.52 Hz, 2H), 8.45 (s, 1H). ^{13}C NMR

(100 MHz, $CDCl_3$): δ 55.69, 60.58, 97.71, 125.54, 129.68, 130.53, 131.64, 132.00, 147.86, 153.16, 157.80. HRMS: found 350.0382 (M^++H); $C_{16}H_{17}{}^{80}BrNO_3$ requires 350.0392.

(*E*)-1-(4-Nitrophenyl)-*N*-(3,4,5-trimethoxyphenyl)methanimine (5d). Preparation as described above from 4-nitrobenzaldehyde and 3,4,5-trimethoxyaniline. The product was obtained as light yellow solid, yield 90%, Mp 160-162 ˚C. [3] IR (KBr) ν_{max}: 1630 (C=N) cm^{-1}. ^1H NMR (400 MHz, $CDCl_3$): δ 3.90 (s, 3H), 3.94 (s, 6H), 6.57 (s, 2H), 8.09 (d, J = 9.04 Hz), 8.35 (d, J = 8.56 Hz), 8.59 (s, 1H). ^{13}C NMR (100 MHz, $CDCl_3$): δ 55.73, 60.60, 97.98, 123.61, 123.87, 128.88, 136.92, 141.02, 146.19, 148.80, 153.25, 156.04. HRMS: found 317.1146 (M^++H); $C_{16}H_{17}N_2O_5$ requires 317.1137.

(*E*)-*N*,*N*-Dimethyl-4-(((3,4,5-trimethoxyphenyl)imino)methyl)aniline (5e). Preparation as described above from 4-(dimethylamino)benzaldehyde and 3,4,5-trimethoxyaniline. The product was obtained as light yellow solid, yield 59%, Mp 97-98 ˚C. [4] IR (KBr) ν_{max}: 1604 (C=N) cm^{-1}. ^1H NMR (400 MHz, $CDCl_3$): δ 3.09 (s, 6H), 3.88 (s, 3H), 3.92 (s, 6H), 6.51 (s, 2H), 6.76 (d, J = 8.80 Hz, 2H), 7.80 (br s, 2H), 8.35 (s, 1H). ^{13}C NMR (100 MHz, $CDCl_3$): δ 39.74, 55.64, 60.57, 97.62, 111.11, 123.50, 130.07, 135.19, 142.97, 152.11, 153.03, 159.20. HRMS: found 315.1710 (M^++H); $C_{18}H_{23}N_2O_3$ requires 315.1709.

(*E*)-1-Phenyl-*N*-(3,4,5-trimethoxyphenyl)methanimine (5f). Preparation as described above from benzaldehyde and 3,4,5-trimethoxyaniline. The product was obtained as pale yellow solid, yield 59%.[5] Mp: 90-92 ˚C. IR (KBr) ν_{max}: 1633 (C=N) cm^{-1}. ^1H NMR (400 MHz, $CDCl_3$): δ 3.89 (s, 3H), 3.93 (s, 6H), 6.53 (s, 2H), 7.51-7.52 (m, 3H), 7.92-7.94 (m, 2H), 8.50 (s, 1H). ^{13}C NMR (100 MHz, $CDCl_3$): δ 55.69, 60.58, 97.70, 128.36, 128.38, 129.31, 131.04, 135.51, 135.96, 153.12, 159.34. HRMS: found 272.1284 (M^++H); $C_{16}H_{18}NO_3$ requires 272.1287.

(*E*)-1-*p*-Tolyl-*N*-(3,4,5-trimethoxyphenyl)methanimine (5g). Preparation as described above from 4-methylbenzaldehyde and 3,4,5-trimethoxyaniline. The product was obtained as pale yellow solid, yield 73%, Mp 107-108 ˚C. [6] IR (KBr) ν_{max}: 1638 (C=N) cm^{-1}. ^1H NMR (400 MHz, $CDCl_3$): δ 2.44 (s, 3H), 3.88 (s, 3H), 3.92 (s, 6H), 6.51 (s, 2H), 7.30 (d, J = 8.04 Hz, 2H), 7.81 (d, J = 8.04 Hz, 2H), 8.46 (s, 1H). ^{13}C NMR (100 MHz, $CDCl_3$): δ 21.21, 55.67, 60.57, 97.68, 128.35, 129.12, 132.96, 135.78, 141.57, 147.60, 153.09, 159.31. HRMS: found 286.1449 (M^++H); $C_{17}H_{20}NO_3$ requires 286.1443.

(*E*)-1-(4-Methoxyphenyl)-*N*-(3,4,5-trimethoxyphenyl)methanimine (5h). Preparation as described above from 4-methoxybenzaldehyde and 3,4,5-trimethoxyaniline. The product was obtained as pale yellow solid, yield 88%, Mp 114-115 ˚C [7]. IR (KBr) ν_{max}: 1607 (C=N) cm^{-1}. ^1H NMR (400 MHz, $CDCl_3$): δ 3.88 (s, 3H), 3.90 (s, 3H), 3.92 (s, 6H), 6.49 (s, 2H), 7.01 (d, J = 7.80 Hz, 2H), 7.86 (d, J = 7.84 Hz, 2H), 8.42 (s, 1H). ^{13}C NMR (100 MHz, $CDCl_3$): δ 55.00, 55.66, 60.57, 97.63, 113.77, 128.61, 130.03, 135.61, 147.85, 153.08, 158.68, 161.83. HRMS: found 302.1400 (M^++H); $C_{17}H_{20}NO_4$ requires 302.1392.

(*E*)-1-(4-Ethoxyphenyl)-*N*-(3,4,5-trimethoxyphenyl)methanimine (5i). Preparation as described above from 4-ethoxybenzaldehyde and 3,4,5-trimethoxyaniline. The product was obtained as pale yellow solid, yield 71%, Mp: 103-105 ˚C. [4] IR (KBr) ν_{max}: 1608 (C=N) cm^{-1}. ^1H NMR (400 MHz, $CDCl_3$): δ 1.47 (t, J = 7.04Hz, 3H), 3.88 (s, 3H), 3.92 (s, 6H), 4.13 (q, J = 7.02 Hz, 2H), 6.51 (s, 2H), 6.99 (d, J = 9.00 Hz, 2H), 7.87 (d, J = 7.04 Hz, 2H), 8.42 (s, 1H). ^{13}C NMR (100 MHz, $CDCl_3$): δ 14.76, 56.13, 61.04, 63.72, 98.11, 114.74, 128.69, 130.68, 132.03, 136.14, 153.56, 159.23, 160.09. HRMS: found 316.1551 (M^++H); $C_{18}H_{22}NO_4$ requires 316.1549.

(*E*)-1-(Naphthalen-1-yl)-*N*-(3,4,5-trimethoxyphenyl)methanimine (5m). Preparation as described above from 1-naphthaldehyde and 3,4,5-trimethoxyaniline. The product was obtained as yellow solid, yield 80%, Mp 110-112 ˚C. [3] IR (KBr) ν_{max}: 1637 (C=N) cm^{-1}. ^1H NMR (400 MHz, $CDCl_3$): δ 3.92 (s, 3H), 3.96 (s, 6H), 6.60 (s, 2H), 7.59-8.13 (m, 7H), 9.14 (s, 1H). ^{13}C NMR (100 MHz, $CDCl_3$): δ 56.21, 61.09, 98.22, 124.20, 124.92, 125.34, 126.31, 128.85, 129.90, 132.06, 133.94, 135.35, 136.75, 148.59, 153.67, 159.51. HRMS: found 322.1436 (M^++H); $C_{20}H_{20}NO_3$ requires 322.1443.

(*E*)-1-(Naphthalen-2-yl)-*N*-(3,4,5-trimethoxyphenyl)methanimine (5n). Preparation as described above from 2-naphthaldehyde and 3,4,5-trimethoxyaniline. The product was obtained as yellow solid, yield 78%, Mp 122-124 ˚C. [4]. IR (KBr) ν_{max}: 1637 (C=N) cm^{-1}. ^1H NMR (400 MHz, $CDCl_3$): δ 3.90 (s, 3H), 3.95 (s, 6H), 6.63 (s, 2H), 7.59-8.29 (m, 7H) 8.68 (s, 1H). ^{13}C NMR (100 MHz, $CDCl_3$): δ 56.17, 61.07, 98.25, 126.71, 127.66, 128.11, 128.77, 128.80, 129.15, 133.11, 134.12, 135.06, 136.49, 147.90, 153.63, 159.75. HRMS: found 322.1438 (M^++H); $C_{20}H_{20}NO_3$ requires 322.1443.

(*E*)-1-(3-((*tert*-Butyldimethylsilyl)oxy)-4-methoxyphenyl)-*N*-(3,4,5-trimethoxy phenyl) methanimine (5o). Preparation as described above from 3-(*tert*-

butyldimethylsilanyloxy)-4-methoxybenzaldehyde and 3,4,5-trimethoxyaniline. The product was obtained as amber solid, yield 90%. [7] Mp 85-86 ˚C. IR (KBr) ν_{max}: 1625 (C=N) cm^{-1}. ^{1}H NMR (400 MHz, $CDCl_3$): δ 0.20 (s, 6H), 1.03 (s, 9H), 3.87 (s, 3H), 3.89 (s, 3H), 3.91 (s, 6H), 6.50 (s, 1H), 6.94 (d, J = 8.52 Hz, 1H), 7.47 (m, 2H), 8.35 (s, 1H). ^{13}C NMR (100 MHz, $CDCl_3$): δ -5.05, 17.96, 25.18, 25.25, 55.01, 55.67, 60.54, 97.73, 111.04, 119.96, 125.82, 128.62, 129.75, 144.92, 147.32, 153.08, 153.83, 158.84. HRMS: found 431.2127 (M^+); $C_{23}H_{33}NO_5Si$ requires 431.2128.

(*E*)-1-(4-Methoxy-3-nitrophenyl)-*N*-(3,4,5-trimethoxyphenyl)methanimine (5p). Preparation as described above from 4-methoxy-3-nitrobenzaldehyde and 3,4,5-trimethoxyaniline. The product was obtained as yellow solid, yield 84%, Mp 166-167 ˚C. [7] IR (KBr) ν_{max}: 1615 (C=N) cm^{-1}. ^{1}H NMR (400 MHz, $CDCl_3$): δ 3.89 (s, 3H), 3.93 (s, 6H), 4.06 (s, 3H), 6.52 (s, 2H), 7.21 (d, J = 8.52 Hz, 1H), 8.12-8.15 (m, 1H), 8.40 (d, J = 2.00 Hz, 1H), 8.45 (s, 1H). ^{13}C NMR (100 MHz, $CDCl_3$): δ 55.69, 56.39, 60.58, 97.72, 113.20, 125.57, 128.48, 133.33, 136.28, 139.37, 146.55, 153.17, 154.41, 155.67. HRMS: found 347.1234 (M^++H); $C_{17}H_{19}N_2O_6$ requires 347.1243.

(*E*)-1-(4-(Methylthio)phenyl)-*N*-(3,4,5-trimethoxyphenyl)methanimine (5q). Preparation as described above from 4-methylsulfanylbenzaldehyde and 3,4,5-trimethoxyaniline. The product was obtained as yellow solid, yield 88%, 2.79 g, Mp 96 ^{o}C. [3] IR (KBr) ν_{max}: 1628 cm^{-1} (C=N); ^{1}H NMR (400 MHz, $CDCl_3$): δ2.52 (s, 3H), 3.87 (s, 3H), 3.89 (s, 6H), 6.48-7.80 (m, 6H), 8.41 (s, 1H). ^{13}C NMR (100 MHz, $CDCl_3$): δ14.57, 55.65, 60.55, 97.69, 125.16, 132.19, 135.83, 142.87, 147.51, 153.09, 158.51. HRMS: found 318.1162 (M^++H); $C_{17}H_{20}NO_3S$ requires 318.1164.

4-[(3,4,5-Trimethoxyphenylimino)methyl]benzonitrile (5r). Preparation as described above from 4-formylbenzonitrile and 3,4,5-trimethoxyaniline. The product was obtained as pale yellow solid, yield 2.72 g, 92%. [3] Mp 134 ^{o}C. IR (KBr) ν_{max}: 1580 cm^{-1} (C=N), 2223 cm^{-1} (C≡N). ^{1}H NMR (400 MHz, $CDCl_3$): δ 3.88 (s, 3H), 3.92 (s, 6H), 6.55-8.02 (m, 6H), 8.53 (s, 1H); ^{13}C NMR (100 MHz, $CDCl_3$): δ 56.18, 61.05, 98.36, 114.34, 118.48, 129.05, 132.58, 137.20, 139.85, 146.76, 153.68, 157.07. HRMS: found 297.1250 (M^++H); $C_{17}H_{17}N_2O_3$ requires 297.1239.

(*E*)-N-(3,5-Dimethoxyphenyl)-1-(4-methoxyphenyl)methanimine (5s). Preparation as described above using the general method III above and was obtained from 4-methoxybenzaldehyde and 3,5-dimethoxyaniline as an oil. [8] Yield: 97%, Purity (HPLC): 92%. IRνmax (ATR): 1592.2 cm^{-1}(C=N). ^{1}H NMR (400 MHz, $CDCl_3$): δ 3.79 (s, 6 H), 3.84 (s, 3 H), 6.30 - 6.36 (m, 3 H), 6.95 (d, *J* = 7.93 Hz, 2 H), 7.81 (d, *J* = 7.93

Hz, 2 H), 8.35 (s, 1 H). ^{13}C NMR (100 MHz, $CDCl_3$): δ 55.37, 55.52, 97.92, 98.97, 114.15, 114.26, 129.00, 130.54, 131.92, 154.52, 159.83. HRMS: found 272.1289 (M^++H); $C_{16}H_{18}NO_3$ requires 272.1287.

(*E*)-*N*-(4-Fluorophenyl)-1-(3,4,5-trimethoxyphenyl)methanimine (6a). Preparation as described above from 3,4,5-trimethoxybenzaldehyde and 4-fluoroaniline. The product was obtained as colourless solid, yield 65%, Mp 110-113 ˚C. [9] IR (KBr) ν_{max}: 1620 (C=N) cm^{-1}. ^1H NMR (400 MHz, $CDCl_3$): δ 3.94 (s, 3H), 3.96 (s, 6H), 7.07-7.12 (m, 2H), 7.16 (d, J = 7.52 Hz, 2H), 7.19-7.22 (m, 2H), 8.35 (s, 1H). ^{13}C NMR (100 MHz, $CDCl_3$): δ 55.79, 60.55, 105.25, 115.32, 115.55, 121.79, 131.06, 140.53, 147.42, 153.07, 159.25, 161.94. HRMS: found 290.1181 (M^++H); $C_{16}H_{17}FNO_3$ requires 290.1192.

(*E*)-*N*-(4-Chlorophenyl)-1-(3,4,5-trimethoxyphenyl)methanimine (6b). Preparation as described above from 3,4,5-trimethoxybenzaldehyde and 4-chloroaniline. The product was obtained as pale green solid (yield 77%); mp: 136-137 ˚C. [10] IR (KBr) ν_{max}: 1625 cm^{-1} (C=N). ^1H NMR (400 MHz, $CDCl_3$): δ 3.94 (s, 3H), 3.97 (s, 6H), 7.18 (br s, 4H), 7.37 (d, J = 8.04 Hz, 2H), 8.35 (s, 1H); ^{13}C NMR (100 MHz, $CDCl_3$): δ 55.84, 60.59, 105.57, 121.76, 128.66, 128.84, 131.25, 131.89, 144.58, 147.20, 153.08, 159.83. HRMS: found 306.0902 (M^++H); $C_{16}H_{17}{}^{35}ClNO_3$ requires 306.0897.

(*E*)-*N*-(4-Bromophenyl)-1-(3,4,5-trimethoxyphenyl)methanimine (6c). Preparation as described above from 3,4,5-trimethoxybenzaldehyde and 4-bromoaniline. The product was obtained as colourless crystals, yield 90%, Mp 140-141 ˚C. [11] IR (KBr) ν_{max}: 1625 (C=N) cm^{-1}. ^1H NMR (400 MHz, $CDCl_3$): δ 3.94 (s, 3H), 3.96 (s, 6H), 7.11 (d, J = 8.52 Hz, 2H), 7.17 (s, 2H), 7.52 (d, J = 8.52 Hz, 2H), 8.34 (s, 1H). ^{13}C NMR (100 MHz, $CDCl_3$): δ 55.82, 60.57, 105.45, 118.86, 122.14, 131.25, 131.53, 131.77, 140.78, 153.08, 153.19, 159.84. HRMS: found 350.0385 (M^++H); $C_{16}H_{17}{}^{79}BrNO_3$ requires 350.0392.

(*E*)-*N*-Phenyl-1-(3,4,5-trimethoxyphenyl)methanimine (6f). Preparation as described above from 3,4,5-trimethoxybenzaldehyde and aniline. The product was obtained as yellow crystal, yield 98%, Mp 90-92 ˚. [7] IR (KBr) ν_{max}: 1629 (C=N) cm^{-1}. ^1H NMR (400 MHz, $CDCl_3$): δ 3.94 (s, 3H), 3.96 (s, 6H), 7.19 (s, 2H), 7.24-7.27 (m, 3H), 7.39-7.43 (m, 2H), 8.37 (s, 1H). ^{13}C NMR (100 MHz, $CDCl_3$): δ 55.81, 60.55, 105.35, 120.43, 125.51, 128.74, 131.16, 140.53, 151.36, 153.07, 159.49. HRMS: found 272.1284 (M^++H); $C_{16}H_{18}NO_3$ requires 272.1287.

(*E*)-*N*-*p*-Tolyl-1-(3,4,5-trimethoxyphenyl)methanimine (6g). Preparation as described above from 3,4,5-trimethoxybenzaldehyde and *p*-toluidine. The product was obtained as colourless crystals, yield 69%, Mp 100-101 ˚C. [12] IR (KBr) ν_{max}: 1622 (C=N) cm^{-1}. ^{1}H NMR (400 MHz, $CDCl_3$): δ 2.40 (s, 3H), 3.94 (s, 3H), 3.97 (s, 6H), 7.15-7.23 (m, 6H), 8.39 (s, 1H). ^{13}C NMR (100 MHz, $CDCl_3$): δ 20.59, 55.84, 60.57, 105.38, 120.36, 129.37, 130.26, 131.59, 139.98, 144.67, 153.04, 158.72. HRMS: found 286.1443 (M^++H); $C_{17}H_{20}NO_3$ requires 286.1443.

(*E*)-*N*-(4-Ethylphenyl)-1-(3,4,5-trimethoxyphenyl)methanimine (6h). Preparation as described above from 3,4,5-trimethoxybenzaldehyde and 4-ethylaniline. The product was obtained as pale yellow crystals, yield 52%, Mp 85-86 ˚C. [13] IR (KBr) ν_{max}: 1625 (C=N) cm^{-1}. ^{1}H NMR (400 MHz, $CDCl_3$): δ 1.28 (t, J = 7.52 Hz, 3H), 2.70 (q, J = 7.52 Hz, 2H), 3.94 (s, 3H), 3.97 (s, 6H), 7.18-7.20 (m, 4H), 7.25 (d, J = 8.04 Hz, 2H), 8.39 (s, 1H). ^{13}C NMR (100 MHz, $CDCl_3$): δ 15.22, 27.98, 55.82, 60.55, 105.28, 120.42, 128.15, 131.25, 140.41, 141.83, 149.01, 153.05, 158.71. HRMS: found 300.1597 (M^++H); $C_{18}H_{22}NO_3$ requires 300.1600.

(*E*)-*N*-(4-Methoxyphenyl)-1-(3,4,5-trimethoxyphenyl)methanimine (6i). Preparation as described above from 3,4,5-trimethoxybenzaldehyde and 4-methoxyaniline. The product was obtained as pale green solid, yield 83%, Mp 109-111 ˚C. [7] IR (KBr) ν_{max}: 1619 (C=N) cm^{-1}. ^{1}H NMR (400 MHz, $CDCl_3$): δ 3.85 (s, 3H), 3.94 (s, 3H), 3.97 (s, 6H), 6.95 (d, J = 8.52 Hz, 2H), 7.19 (s, 2H), 7.27 (d, J = 8.52 Hz, 2H), 8.40 (s, 1H). ^{13}C NMR (100 MHz, $CDCl_3$): δ 55.06, 55.82, 60.56, 105.20, 113.95, 121.73, 129.04, 140.95, 143.90, 153.04, 157.56, 157.85. HRMS: found 302.1390 (M^++H); $C_{17}H_{20}NO_4$ requires 302.1392.

***N*-((*E*)-4-(3,4,5-Trimethoxybenzylideneamino)phenyl)acetamide (6j).** Preparation as described above from 3,4,5-trimethoxybenzaldehyde and 4-aminoacetanilide. The product was obtained as yellow solid, yield 38%, Mp 161-162 ˚C. [14] IR (KBr) ν_{max}: 1667 (C=O), 1621 (C=N) cm^{-1}. ^{1}H NMR (400 MHz, $CDCl_3$): δ 2.20 (s, 3H), 3.93 (s, 3H), 3.95 (s, 6H), 7.16 (s, 2H), 7.22 (d, J = 8.52 Hz), 7.52 (s, 1H), 7.56 (d, J = 9.04 Hz, 2H), 8.37 (s, 1H). ^{13}C NMR (100 MHz, $CDCl_3$): δ 24.14, 55.78, 60.55, 105.18, 120.20, 121.09, 121.81, 131.27, 135.66, 140.99, 147.44, 153.04, 158.78, 167.93. HRMS: found 329.1510 (M^++H); $C_{18}H_{21}N_2O_4$ requires 329.1501.

(*E*)-N-(4-(Methylthio)phenyl)-1-(3,4,5-trimethoxyphenyl)methanimine (6k). Preparation as described above from 3,4,5-trimethoxybenzaldehyde and 4-methylthioaniline. The product was obtained as yellow crystals, Yield: 92%, Mp 112

°C. [13] IR (NaCl, film) ν_{max}: 1677.89 cm^{-1}(C=N). ^{1}H NMR (400 MHz,$CDCl_3$): δ ppm 2.48 (s, 3H), 3.86 – 3.96 (m, 9 H), 7.14 (d, *J*=8.54 Hz, 2 H), 7.23-7.28 (m, 2 H), 7.12 (s, 2H), 8.33 (s, 1 H). ^{13}C NMR (100 MHz, $CDCl_3$): δ ppm 16.34, 56.23, 60.96, 105.71, 121.50, 127.66, 131.69, 135.80, 140.95, 149.17, 153.49, 159.18. HRMS: found 340.0994 (M^{+}+Na); $C_{17}H_{19}NO_3SNa$ requires 340.0983.

Table S1: Tier-1 Profiling Screen of Selected β-Lactams[a]

Molecule	ADMET Solubility[b]	ADMET Solubility Level[c]	ADMET BBB[d]	ADMET BBB Level[e]	ADMET EXT CYP2D6 Prediction	ADMET EXT Hepatotoxic Prediction
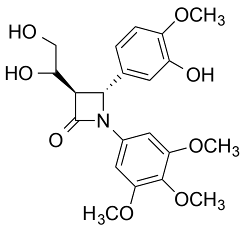 13	-2.32	3	-	4	false	true
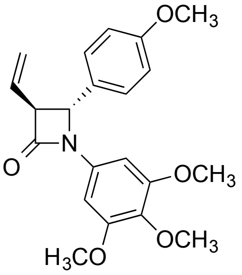 7h	-4.27	2	-0.07	2	false	false
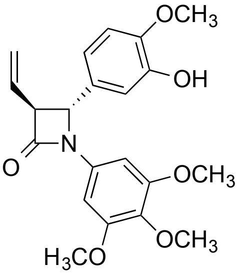 7s	-3.83	3	-0.47	2	false	true
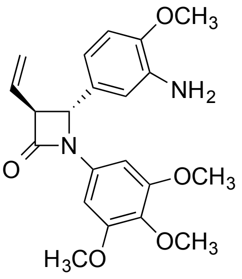 7t	-4.07	2	-0.72	3	false	false

Molecule	ADMET Solubility[b]	ADMET Solubility Level[c]	ADMET BBB[d]	ADMET BBB Level[e]	ADMET EXT CYP2D6 Prediction	ADMET EXT Hepatotoxic Prediction
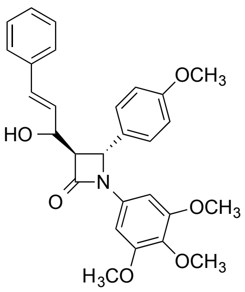 10f	-4.55	2	-0.11	2	false	false
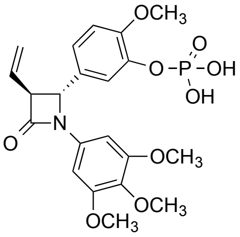 17b	-3.81	3	-	4	false	true

[a]Calculated using Pipeline Pilot Professional (v8.5.0.200) BIOVIA, Dassault Systèmes
[b]ADMET Solubility: Log of the water solubility at 25 °C (LogSw)(mol/L)
[c]ADMET Solubility Level: Ranking of the solubility values into the following classes: 0: Extremely Low; 1: Very Low; 2: Low; 3: Good; 4: Optimal; 5: Very Soluble
[d]ADMET BBB: Predicts the blood brain barrier penetration of a molecule, defined as the ratio of the concentrations of solute (compound) on the both sides of the membrane after oral administration.
[e]ADMET Blood Brain Barrier Absorption (BBB) Level: Ranking of LogBBB values into one of the following levels: 0: Very High; 1: High; 2: Medium; 3: Low; 4: Undefined (molecule is outside the confidence area of the regression model used to calculate LogBB)

Table S2: ADMET and Lipinski Properties for Selected β-Lactams[a]

Molecule	ADMET Absorption Level[b]	ADMET EXT PPB Prediction[c]	ALogP	MW	HA	HD	RB	Volume	PSA
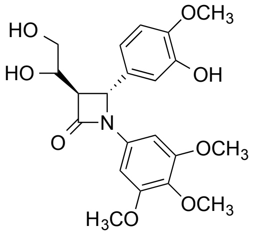 13	0	false	1.26	419.43	8	3	8	274.74	117.92
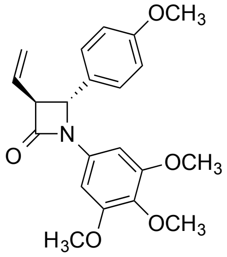 7h	0	true	3.17	369.41	5	0	7	250.38	57.230
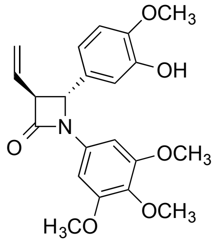 7s	0	true	2.92	385.41	6	1	7	258.96	77.460
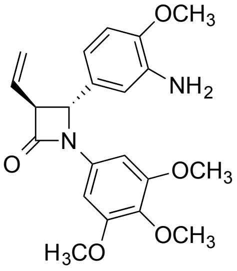 7t	0	true	2.42	384.43	6	1	7	260.33	83.250

Molecule	ADMET Absorption Level[b]	ADMET EXT PPB Prediction[c]	ALogP	MW	HA	HD	RB	Volume	PSA
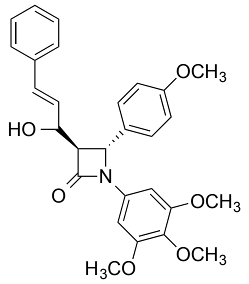 10f	0	true	4.09	475.53	6	1	9	322.41	77.460
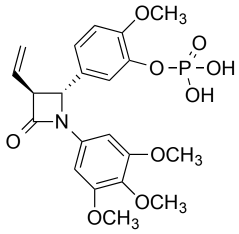 17b	1	true	2.67	465.39	9	2	9	291.89	133.80

[a]Calculated using Pipeline Pilot Professional (v8.5.0.200) BIOVIA, Dassault Systèmes
[b]ADMET Absorption Level: Ranking of the molecule into one of the following levels: 0: Good; 1: Moderate; 2: Poor; 3: Very Poor
[c]ADMET Plasma Protein Binding (PPB) Prediction: If true, the compound is predicted to be a binder (>=90%). Otherwise, it is predicted to be a weak or nonbinder(<90%).

Table S3: Comparative Antitumour Evaluations of compounds **7h, 7s, 7t, 17b, 17c** in the NCI60 ***Leukaemia, Non-Small Cell Lung Cancer***, ***Colon Cancer*** and ***CNS Cancer*** cell lines *in vitro* primary screen[a]

**Cell line**	**7h GI50 (µM)**	**7s GI50 (µM)**	**7t GI50 (µM)**	**17b GI50 (µM)**	**17c GI50 (µM)**
Leukemia					
CCRF-CEM	Nt[b]	nt[a]	0.0326	0.0604	0.0325
HL-60(TB)	nt[a]	nt[a]	0.0137	0.0273	0.0288
K-562	nt[a]	nt[a]	0.0372	0.0415	0.035
MOLT-4	nt[a]	nt[a]	0.04	0.129	0.0477
RPMI-8226	nt[a]	nt[a]	<0.0100	0.0802	0.0482
SR	nt[a]	nt[a]	0.0439	0.0572	0.0335
Non-Small Cell Lung Cancer					
A549/ATCC	0.0413	<0.0100	0.0392	0.0458	0.0382
EKVX	0.0583	0.0258	nt[a]	nt[b]	nt[b]
HOP-62	0.0369	<0.0100	0.0412	0.09	0.024
HOP-92	nt[b]	nt[b]	0.0393	1.78	0.0979
NCI-H226	0.0339	<0.0100	0.0387	>100	>100
NCI-H23	0.0245	<0.0100	0.0387	0.0637	0.0444
NCI-H332M	Nt[b]	0.0855	0.0645	0.0903	0.0449
NCI-H460	0.036	0.0131	0.0355	0.0386	0.0352
NCI-H552	0.0206	<0.0100	<0.0100	0.0227	0.0142
Colon Cancer					
COLO 205	0.0206	0.0321	0.0175	1.91	0.204
HCT-2998	0.0353	0.0364	0.0368	0.19	0.0409
HCT-116	0.0307	<0.0100	0.0276	0.0439	0.035
HCT-15	0.0371	0.018	0.0287	0.0474	0.0296
HT29	0.0337	0.429	0.0179	2.78	0.356
KM12	0.0351	<0.0100	0.0242	0.0391	0.022
SW-620	0.0402	<0.0100	0.0405	0.0552	0.0379
CNS Cancer					
SF-268	0.0476	<0.0100	0.0546	0.0884	0.0433
SF295	0.0358	0.271	0.0213	0.0291	0.0243
SF539	nt[b]	Nt[b]	<0.0100	0.042	0.0162
SNB-19	0.0542	0.0405	0.049	0.459	0.0639
SNB-75	0.0372	0.0153	0.0301	0.0365	0.0237
U251	0.0342	0.0156	0.0362	0.0343	0.0313
Prostate cancer					
PC-3	0.0374	<0.0100	0.0415	0.0536	0.0349
DU-145	0.0298	<0.0100	0.0365	0.0596	0.0413

[a] GI_{50} is the mean concentrations required to cause 50% reduction in proliferation in the growth of the cancer cells over the total NCI 60 cell line panel in the assay [15].
[b]nt = not tested;

Table S4: Comparative Antitumour Evaluations of compounds **7h, 7s, 7t, 17b, 17c** in the NCI60 ***Melanoma***, ***Ovarian cancer***, ***Renal cancer*** and ***Breast cancer*** cell line *in vitro* primary screen[a]

**Cell line**	**7h** **GI_{50}** **(μM)[a]**	**7s** **GI_{50}** **(μM) [a]**	**7t** **GI_{50}** **(μM) [a]**	**17b** **GI_{50}** **(μM) [a]**	**17c** **GI_{50}** **(μM)[a]**
Melanoma					
LOX IMVI	0.0416	<0.0100	<0.0100	0.0422	0.0241
MALME-3M	0.073	0.0283	>100	54.5	71.4
M14	0.0252	<0.0100	0.0272	0.748	0.0317
MDA-MB-435	0.0226	<0.0100	<0.0100	0.021	0.0128
SK-MEL-2	0.191	0.0314	0.0656	0.0858	0.0544
SK-MEL-28	0.0599	<0.0100	0.0181	0.0977	7.46
SK-MEL-5	0.0194	<0.0100	0.0146	0.0312	0.019
UACC-257	42.2	>100	>100	>100	>100
UACC-62	0.0502	<0.0100	12.3	0.0511	0.0348
Ovarian cancer					
IGROV1	0.0404	<0.0100	0.0496	0.0669	0.0376
OVCAR-3	0.0251	<0.0100	0.0181	0.0497	0.0368
OVCAR-4	0.0776	0.0131	<0.0100	0.16	0.168
OVCAR-5	0.0587	0.0453	0.0446	3.57	0.444
OVCAR-8	Nt[b]	Nt[b]	0.0148	0.0442	0.0327
NCI/ADR-RES	0.0272	<0.0100	<0.0100	0.037	0.0191
SK-OV-3	0.0285	0.0105	0.0457	0.0977	0.0341
Renal cancer					
786-0	0.0339	0.0273	0.0346	0.229	0.0445
A498	0.0183	<0.0100	0.011	0.0218	0.0129
ACHN	0.0615	<0.0100	0.0373	0.0645	0.0385
CAKI-1	0.392	0.0402	0.0329	0.0717	0.0352
RXF 393	0.0287	0.0136	0.0366	0.0648	0.0369
SN12C	0.0552	<0.0100	nt[a]	0.146	0.0548
TK-10	Nt[b]	0.0102	26.8	11.4	0.0736
UO-31	0.0709	0.0111	0.064	0.254	0.0426
Breast cancer					
MCF-7	0.0306	<0.0100	<0.0100	0.0394	0.0251
MDA-MB-231/ATCC	0.0317	<0.0100	0.0415	0.0486	0.031
HS 578T	0.0727	<0.0100	0.0398	0.0531	0.0371
BT-549	0.0325	<0.0100	0.0259	0.115	0.0338
T-47D	20.5	0.0299	nt[a]	>100	>100
MDA-MB-468	0.0227	<0.0100	<0.0100	0.0496	0.0276

[a] GI_{50} is the mean concentrations required to cause 50% reduction in proliferation in the growth of the cancer cells in the assay [15].
[b]nt = not tested.

Table S5: NCI 60 cell line mean screening results for selected compounds

NCI Ref. No	Compound number	GI_{50} (µM)[a]	TGI (µM)[b]	LC_{50} (µM)[a]
**S-613729**	**CA-4**	0.0993	10.30	85.50
**S-762036**	**7h**	0.0525	23.44	75.86
**S-762031**	**7s**	0.0229	5.89	74.13
**S-775039**	**7t**	0.0479	50.12	89.12
**S-775042**	**17a**	0.1412	39.8	87.09
**S-775043**	**17b**	0.0724	36.30	87.09
**S-775044**	**17c**	0.1380	43.65	85.11

[a] GI_{50} and LC_{50} are the mean concentrations required to inhibit the growth and kill 50 % of the cells over the total NCI 60 cell line panel in the assay respectively [15].
[b] TGI is the mean concentration required to completely inhibit the growth of all cells over the total NCI 60 cell line panel [15].

Table S6: Standard COMPARE analysis of β-lactam **7h**

Rank	Compound	r
	Based on GI_{50} mean graph	
1	Vincristine sulfate (hiConc = 10-5 M)	0.623
2	Maytansine	0.511
3	Tiazofurin	0.477
4	Vincristine sulfate (hiConc = 10-3 M)	0.458
5	Vinblastine sulfate (hiConc = 10-4 M)	0.453
	Based on TGI mean graph	
1	Vinblastine sulfate (hiConc = 10-4 M)	0.567
2	Maytansine	0.565
3	Vinblastine sulfate (hiConc = 10-5.6	0.552
4	M) Vincristine sulfate (hiConc = 10-5	0.538
5	M) A-TGDR	0.479
	Based on LC_{50} mean graph	
1	Tetraplatin	0.886
2	B-TGDR	0.879
3	Didemnin B	0.862
4	Paclitaxel (Taxol)	0.765
5	Maytansine	0.743

The target set was the standard agent database and the target set endpoints were selected to be equal to the seed end points. Standard COMPARE analysis was performed. Correlation values (r) are Pearson correlation coefficients. Vincristine sulfate appears at different concentrations as it has been tested by the NCI at multiple concentration ranges [15].

Table S7: Standard COMPARE analysis of β-lactam **7s**

Rank	Compound	r
	Based on GI_{50} mean graph	
1	Vincristine sulfate (hiConc = 10-5 M)	0.471
2	Maytansine	0.436
3	Glycoxalic acid	0.413
4	Vincristine sulfate (hiConc = 10-3 M)	0.401
5	DHAD (Mitoxantrone)	0.388
	Based on TGI mean graph	
1	Vinblastine sulfate (hiConc = 10-5.6 M)	0.665
2	Maytansine	0.644
3	Vincristine sulfate (hiConc = 10-3 M)	0.594
4	Vinblastine sulfate (hiConc = 10-4 M)	0.564
5	Vincristine sulfate (hiConc = 10-5 M	0.550
	Based on LC_{50} mean graph	
1	*5-FUDR*	0.769
2	*Tetraplatin*	0.714
3	*Cisplatin*	0.694
4	Maytansine	0.688
5	Rhizoxin	0.686

The target set was the standard agent database and the target set endpoints were selected to be equal to the seed end points. Standard COMPARE analysis was performed. Correlation values (r) are Pearson correlation coefficients. Vincristine sulfate appears at different concentrations as it has been tested by the NCI at multiple concentration ranges [15]

Table S8: Toxicity at 10 μM against two concentrations of murine epithelial cells.

	**% Viable cells (10 μM, 25,000 cells/mL)**	**% Viable cells (10 μM, 50,000 cells/mL)**
**CA-4**	58.0	75.0
**7s**	60.0	73.3

Table S9. Toxicity at IC_{50} against two concentrations of murine epithelial cells.

	**IC_{50}, MCF-7 (nM)**	**% Viable cells at IC_{50} (25,000 cells/mL)**	**% Viable cells at IC_{50} (50,000 cells/mL)**
**CA-4**	5.2	73.6	92.1
**7s**	1.4	93.1	93.2

References

1. Carr, M.; Greene, L.M.; Knox, A.J.; Lloyd, D.G.; Zisterer, D.M.; Meegan, M.J. Lead identification of conformationally restricted beta-lactam type combretastatin analogues: Synthesis, antiproliferative activity and tubulin targeting effects. *Eur J Med Chem* **2010**, *45*, 5752-5766.

2. Elmeligie, S.; Taher, A.T.; Khalil, N.A.; El-Said, A.H. Synthesis and cytotoxic activity of certain trisubstituted azetidin-2-one derivatives as a cis-restricted combretastatin a-4 analogues. *Arch Pharm Res* **2017**, *40*, 13-24

3. Greene, T.F.; Wang, S.; Greene, L.M.; Nathwani, S.M.; Pollock, J.K.; Malebari, A.M.; McCabe, T.; Twamley, B.; O'Boyle, N.M.; Zisterer, D.M.*, et al.* Synthesis and biochemical evaluation of 3-phenoxy-1,4-diarylazetidin-2-ones as tubulin-targeting antitumor agents. *J Med Chem* **2016**, *59*, 90-113.

4. O'Boyle, N.M.; Carr, M.; Greene, L.M.; Keely, N.O.; Knox, A.J.; McCabe, T.; Lloyd, D.G.; Zisterer, D.M.; Meegan, M.J. Synthesis, biochemical and molecular modelling studies of antiproliferative azetidinones causing microtubule disruption and mitotic catastrophe. *Eur J Med Chem* **2011**, *46*, 4595-4607.

5. Georg, G.I.; He, P.; Kant, J.; Mudd, J. N-vinyl and n-unsubstituted beta-lactams from 1-substituted 2-aza-1,3-butadienes. *Tetrahedron Lett* **1990**, *31*, 451-454.

6. Arroyo, Y.; Sanz-Tejedor, M.A.; Alonso, I.; Garcia-Ruano, J.L. Synthesis of optically pure vic-sulfanyl amines mediated by a remote sulfinyl group. *Org Lett* **2011**, *13*, 4534-4537.

7. O'Boyle, N.M.; Carr, M.; Greene, L.M.; Bergin, O.; Nathwani, S.M.; McCabe, T.; Lloyd, D.G.; Zisterer, D.M.; Meegan, M.J. Synthesis and evaluation of azetidinone analogues of combretastatin a-4 as tubulin targeting agents. *J Med Chem* **2010**, *53*, 8569-8584.

8. Tripodi, F.; Pagliarin, R.; Fumagalli, G.; Bigi, A.; Fusi, P.; Orsini, F.; Frattini, M.; Coccetti, P. Synthesis and biological evaluation of 1,4-diaryl-2-azetidinones as specific anticancer agents: Activation of adenosine monophosphate activated protein kinase and induction of apoptosis. *J Med Chem* **2012**, *55*, 2112-2124.

9. Sandhar, R.K.S., J. R.; Manrao, M. R. . Reaction of acetylacetone with benzal-4-fluoroanilines and antifungal potential of the products. *Journal of Indian Council of Chemists* **2005**, *22*, 32-34.

10. Dehno Khalaji, A.; Fejfarova, K.; Dusek, M. N,n'-bis(3,4-dimethoxy-benzyl-idene)butane-1,4-diamine. *Acta Crystallogr Sect E Struct Rep Online* **2009**, *65*, o1773.

11. Khalaji, A.D.; Weil, M.; Gotoh, K.; Ishida, H. 4-bromo-n-(3,4,5-trimethoxy-benzyl-idene)aniline. *Acta Crystallogr Sect E Struct Rep Online* **2009**, *65*, o436.

12. Yang, Z. Synthesis and in vitro biological activity evaluation of the derivatives of combretastatin a-4. *Letters in Drug Design & Discovery* **2006** *3*, 544-546.

13. Cushman, M.; He, H.M.; Lin, C.M.; Hamel, E. Synthesis and evaluation of a series of benzylaniline hydrochlorides as potential cytotoxic and antimitotic agents acting by inhibition of tubulin polymerization. *J Med Chem* **1993**, *36*, 2817-2821.

14. Gaidhane, M.K.G., A. M.; Lanjewar, K. R. . Novel synthesis and antimicrobial activity of novel schiff base derived quinoline and their beta-lactam derivatives. *International Journal of Pharmacy and Pharmaceutical Sciences* **2013**, *5*, 421-426.
15. National Cancer Institute biological testing branch; National Cancer Institute; Bethesda, MD; **2019.** https://dtp.Nci.Nih.Gov/branches/btb/hfa.Html (accessed 10th January 2019).
